# Aphid alarm pheromone mimicry in transgenic *Chrysanthemum morifolium*: insights into the potential of (*E*)-β-farnesene for aphid resistance

**DOI:** 10.3389/fpls.2024.1373669

**Published:** 2024-04-22

**Authors:** Jinjin Li, Hao Hu, Shengjing Ren, Lu Yu, Yuanyuan Luo, Jiawen Li, Tuo Zeng, Manqun Wang, Caiyun Wang

**Affiliations:** ^1^National Key Laboratory for Germplasm Innovation & Utilization of Horticultural Crops, Huazhong Agricultural University, Wuhan, China; ^2^Hubei Insect Resources Utilization and Sustainable Pest Management Key Laboratory, College of Plant Science and Technology, Huazhong Agricultural University, Wuhan, China

**Keywords:** chrysanthemum, specific expression, (*E*)-beta-farnesene, *TcEbFS*, genetic transformation, aphid resistance

## Abstract

(*E*)-β-Farnesene (EBF) serves as the primary component of the alarm pheromone used by most aphid pest species. Pyrethrum (*Tanacetum cinerariifolium*) exhibits tissue-specific regulation of EBF accumulation and release, effectively mimicking the aphid alarm signal, deterring aphid attacks while attracting aphid predators. However, cultivated chrysanthemum (*Chrysanthemum morifolium*), a popular and economically significant flower, is highly vulnerable to aphid infestations. In this study, we investigated the high expression of the pyrethrum EBF synthase (*Tc*EbFS) gene promoter in the flower head and stem, particularly in the parenchyma cells. Subsequently, we introduced the *TcEbFS* gene, under the control of its native promoter, into cultivated chrysanthemum. This genetic modification led to increased EBF accumulation in the flower stem and young flower bud, which are the most susceptible tissues to aphid attacks. Analysis revealed that aphids feeding on transgenic chrysanthemum exhibited prolonged probing times and extended salivation durations during the phloem phase, indicating that EBF in the cortex cells hindered their host-location behavior. Interestingly, the heightened emission of EBF was only observed in transgenic chrysanthemum flowers after mechanical damage. Furthermore, we explored the potential of this transgenic chrysanthemum for aphid resistance by comparing the spatial distribution and storage of terpene volatiles in different organs and tissues of pyrethrum and chrysanthemum. This study provides valuable insights into future trials aiming for a more accurate replication of alarm pheromone release in plants. It highlights the complexities of utilizing EBF for aphid resistance in cultivated chrysanthemum and calls for further investigations to enhance our understanding of this defense mechanism.

## 1 Introduction

Chrysanthemums hold a prominent place in traditional Chinese culture, and their modern cultivated varieties have emerged from extensive crossbreeding with wild relatives worldwide (Zhang et al., 2021). Breeding strategies in hexaploid chrysanthemum predominantly revolve around conventional methods such as hybridization and vegetative propagation of elite lines. However, a limitation of this approach is that the successful combination of all desired traits within a single cultivar relies more on chance than on a deliberate strategy. Most cultivated chrysanthemums are highly susceptible to aphid infestations, resulting in significant economic losses and the excessive use of toxic pesticides as a primary control measure (Saicharan et al., 2019; Zhong et al., 2022). Chrysanthemums are chosen as a host plant by aphids due to their distinctive color and the presence of volatile compounds (Guo et al., 2020). The mechanisms of parthenogenesis and sexual reproduction enable aphids to swiftly elevate their population density, facilitating their proliferation on the chrysanthemum host. Aphids have evolved a specialized life history strategy that enhances their survival on chrysanthemums (Xia et al., 2023). Various strategies for aphid resistance in chrysanthemums extend beyond chemical measures, encompassing modifications to the chrysanthemum epidermal tissue structure, elevation of defense protein content, regulation of phytohormone levels, and attraction of aphid natural predators. Interestingly, chrysanthemum essential oils contain a diverse array of terpenoids, including monoterpenes and sesquiterpenes, which offer potential medicinal and economic value (Sassi et al., 2014; Jiang et al., 2021), however, their defensive functions were still lacking.

Terpenes, a wide range of C5-branched compounds, play crucial roles as defensive compounds in the ecological and physiological systems of plants (Pichersky and Raguso, 2018; Zhou and Pichersky, 2020). They not only protect plants against biotic stresses, such as pathogens and herbivore attacks, but also serve as signals for communication between conspecifics (plant-plant interactions) (Pinto-Zevallos et al., 2013; Boncan et al., 2020; Loreto and D’Auria, 2022). For instance, terpenes can mediate various biotic interactions by acting as cues for foraging herbivores (Bruce and Pickett, 2011) as well as their natural enemies (Alhmedi et al., 2010). In response to harsh environmental conditions, terpene emissions are typically induced or reduced by diverse stimuli, including mechanical wounding (Kikuta et al., 2011), pathogenic infections (Song and Ryu, 2013), herbivore feeding (Miresmailli et al., 2012), and egg deposition (Green et al., 2012; Piesik et al., 2013). Over the past few decades, extensive studies have focused on the genetic regulation, chemistry, biological activities, and metabolic flux of terpene formation and accumulation (Green et al., 2012; Magnard et al., 2015; Zhou and Pichersky, 2020). More recently, researchers have increasingly emphasized the specific localization and regulation of terpene release in plants. Typically, plants restrict the formation and storage of defensive terpenes to specialized structures, such as epidermal glandular trichomes or internal secretory cavities/ducts, to prevent toxicity and fulfill specific ecological functions (Bouwmeester, 2019; Li et al., 2021).

Among the various terpenes, one sesquiterpene volatile, (*E*)-beta-farnesene (EBF), has attracted significant attention. EBF serves as a major component of the alarm pheromone found in numerous aphid species (Zhang et al., 2017), and it can be recognized by odorant receptors on the antennal segment, inducing dispersal behavior among other colony members in response to predator attacks (Vandermoten et al., 2012). Additionally, plants under herbivore attack produce EBF as a semiochemical to repel aphids or attract insect enemies of the herbivores (Francis et al., 2004, 2005; Wang et al., 2021). EBF was initially discovered to be released from specialized foliar trichomes in wild potato as a means to repel aphids (Gibson and Pickett, 1983). However, the ecological function of EBF as an olfactory cue in the plant field, involving aphid repellence and attraction of natural enemies, remains a topic of debate (Vosteen et al., 2016). Previous attempts to engineer plants for aphid resistance by constitutively releasing EBF have encountered challenges due to low emission levels and aphid habituation to this alarm pheromone (de Vos et al., 2010; Kunert et al., 2010; Bruce et al., 2015). In aphids, low levels of EBF are rapidly released in response to vivid attacks by predators, forming emission blocks that are interrupted by periods without EBF emission, thus correlating with the attacked species (Schwartzberg et al., 2008; Joachim and Weisser, 2013; Joachim et al., 2013). In fact, in the presence of danger, only few aphids emit EBF, and the signal is not amplified by further release of the same chemical from other aphids (Hatano et al., 2008). Typically, when an aphid is under attack by a natural predator, the aphid being directly attacked seldom succeeds in escaping and surviving. The prevailing assumption is that while alarm signals serve to warn conspecifics, they may also incur adverse effects on both the emitter and surrounding conspecifics by attracting additional predators. It is now widely accepted that aphids and predators are not easily deceived: aphids only respond to subtle bursts of pure pheromone signal and quickly habituate to constitutive or slowly induced signals from plants. Predators, on the other hand, become confused and inefficient when attracted to plants constantly emitting alarm pheromones without clear cues regarding the relevant tissue and herbivore. These findings highlight the necessity for a more precise mimicry of alarm pheromone release in plants (Bruce et al., 2015). In a previous study, we demonstrated that pyrethrum flowers could mimic aphids by releasing abundant EBF during the early flowering stages, which directly repelled aphids and attracted ladybird beetles as bodyguards. Notably, pure EBF was highly synthesized and accumulated in the cortex cells of the peduncle, which could be ingested by aphids during probing and subsequently released in their honeydew, acting as an auto-alarm (Li et al., 2019). This subtle defensive strategy was achieved through the cortex-specific expression and accumulation of EBF, distinct from the reported storage sites of other glandular trichomes (Gibson and Pickett, 1983; Picaud et al., 2005).

The enzyme (*E*)-beta-farnesene synthase (EbFS) is responsible for converting FPP into EBF and has been isolated and characterized in various plant species, including Douglas fir (Huber et al., 2005), Yuzu (Maruyama et al., 2001), sweet wormwood (Picaud et al., 2005; Yu et al., 2012), black peppermint (Crock et al., 1997; Prosser et al., 2006), Asian peppermint (Yu et al., 2013), and chamomile (Su et al., 2015). In pyrethrum, *TcEbFS* serves as the key gene responsible for EBF biosynthesis, specifically expressed in the cortex cells (Li et al., 2019). The gene sequences of *EbFS* exhibited significant homology within closely related plant species, notably in the Asteraceae family (Li et al., 2019). However, substantial variations in EBF levels were observed across diverse cultivated *Chrysanthemum* species, with distinct expression patterns evident in different plant species (Su et al., 2015; Zhang et al., 2021). These observed distinctions may be attributed to disparities in their respective promoter regions, thereby influencing unique regulatory mechanisms governing EBF production. In this study, we analyzed the expression pattern of the *TcEbFS* promoter in transgenic *Nicotiana tabacum* and *Chrysanthemum morifolium* ‘1581’. EBF is a minor component blended with the abundant terpenes in this *Chrysanthemum* species (Hu et al., 2018). In chrysanthemum, the low level of EBF content and purity may not trigger biological functions on aphids and its enemies. Based on functional analysis of the *TcEbFS* promoter and gene characterization, we performed genetic transformation of the *TcEbFS* gene in chrysanthemum driven by its native promoter to mimic the aphid defense system of pyrethrum. This biotechnological approach offers a potential pest management strategy that eliminates the need for seasonal insecticide applications. Additionally, we thoroughly examined the limitations of this genetic manipulation in chrysanthemum and presented compelling ideas for future advancements to achieve a more precise mimicry of the aphid alarm pheromone in other closely related plant species.

## 2 Materials and methods

### 2.1 Plant and insect materials

Pyrethrum (*Tanacetum cinerariifolium*) plants were cultivated under controlled greenhouse conditions in Wuhan, China, with a temperature of 20 ± 2°C and a 16/8-h light/dark cycle. Tissue culture seedlings of *Nicotiana tabacum* and *Chrysanthemum morifolium* ‘1581’ were maintained in a tissue culture room at a temperature of 25 ± 2°C and a 16/8 h light/dark cycle. Prior to experimentation, transgenic tobacco and chrysanthemum plants were transferred to a climate room with a relative humidity of 60 ± 10% and a temperature of 26 ± 2°C, where they were grown under a photoperiod of 16/8 h light/dark (Li et al., 2019).

Cotton aphids (*Aphis gossypii*) were initially collected from the field and subsequently reared on chrysanthemum ‘1581’ for at least three generations in the greenhouse (25/20°C day/night temperature, 16/8 h light/dark illumination, relative humidity (RH) of 60% – 70%) (Hu et al., 2018). Laboratory bioassays were conducted in a climate room maintained at a temperature of 25 ± 2°C, a 16/8 h light/dark cycle, and a relative humidity of 60-70% (Hu et al., 2018).

### 2.2 Construction of the transformation vector and plant transformation

For plant transformation, we utilized the pBI121 vector containing the *GUS* gene. The construction of the vector involved amplifying a 2024-bp promoter fragment of *TcEbFS* using high-fidelity DNA polymerase and specific primers that included *HindIII* and *BamHI* restriction sites (Fermentas, Thermo Fisher Scientific Inc., USA) along with 15-bp pBI121 vector sequences (proEbFS-121-F and proEbFS-121-R, as shown in **Supplementary Table S2**). The amplified products were then subcloned into the pBI121 vector after digestion with the respective recombinant enzyme, following the instructions provided in the One-step cloning Kit manual (Vazyme Biotech Co, Ltd). The primer sequences used for construct detection (pBI121-F and pBI121-GUS-R) are listed in **Supplementary Table S2**. The resulting construct was named *pBI121-pE::GUS*, while the *pBI121-p35S::GUS* vector, which contained the CaMV35S promoter upstream from the *gusA* reporter gene, was used as a positive control. Untransformed wild-type (WT) plants served as the negative control. Subsequently, we replaced the *GUS* gene with *TcEbFS* using the primers specified in **Supplementary Table S2** (proEbFS-121-EbFS-F and proEbFS-121-EbFS-R) to create the *pBI121-pE::EbFS* vector. The primer sequences used for construct detection (pBI121-F and EbFS-R) are also listed in **Supplementary Table S2**. The *pBI121-pE::GUS*, *pBI121-p35S::GUS*, and *pBI121-pE::EbFS* constructs were individually introduced into the *Agrobacterium tumefaciens EHA105* strain for chrysanthemum transformation and the *GV3101* strain for tobacco transformation. Approximately sixty leaf explants (1 cm² square) were excised from tissue culture seedlings of chrysanthemum (cultivated for 35 days) and tobacco plants (cultivated for one month) for the transformation process (Mao et al., 2013). Subsequently, leaves were sampled from ten independent tobacco plants and fifteen chrysanthemum plants for PCR identification. From the pool of samples, four T0 positive transgenic tobacco plants and six T0 positive transgenic chrysanthemum plants were identified and selected for GUS staining. For both tobacco and chrysanthemum, we ensured the inclusion of at least two independent transgenic plants for subsequent GUS staining and EBF analysis. Two T0 transgenic chrysanthemum lines showing relatively higher *EbFS* gene expression (PEE-2 and PEE-8) were propagated through tissue culture cuttings for subsequent chemical and biological analyses.

### 2.3 GUS staining of transgenic tobacco and chrysanthemum carrying *pBI121-pE::GUS* vector

To investigate the expression pattern of the *GUS* gene, GUS staining was performed on transgenic tobacco and chrysanthemum plants carrying the *pBI121-pE::GUS* vector. Shoots and petioles were collected from at least two independent transgenic tobacco plants, and GUS histochemical staining was conducted following the instructions provided in the *GUS* reporter gene staining kit manual (Sigma-Aldrich). Similarly, flowers (at least three flowers) at different developmental stages were collected from two independent transgenic chrysanthemum plants to assess the promoter activity of the *EbFS* gene in flower organs. The stained tissues were then cross-sectioned, and the samples were observed under a microscope (Olympus BX53).

### 2.4 *EbFS* gene expression of transgenic chrysanthemum carrying *pBI121-pE::EbFS* construct

For the analysis of *EbFS* gene expression in transgenic chrysanthemum plants, total RNA was isolated using Trizol from leaf, flower stem, and flower head tissues at different developmental stages, as well as after mechanical damage treatment. For the mechanical damage treatment, five flowers were selected and subjected to rapid puncturing ten times using entomological pins on the flower stem. Subsequently, the wounded and unwounded flower stems (3 centimeters below the flower head) were cut and immediately flash-frozen in liquid nitrogen. The RNA samples were reverse transcribed as described previously (Li et al., 2019). Real-time gene expression analysis of *EbFS* and the reference gene *CmUBI* was performed on an Applied Biosystems 7500 platform using SYBR Green I with 6-carboxyl-X-rhodamine (ROX) as an internal standard, following the protocol described by Li et al. (Li et al., 2019).

### 2.5 Solvent extraction of secondary metabolites

To determine the terpene content of different flower parts, fresh plant tissues including leaf, flower head, and flower stem were extracted using hexane, and the extracts were subjected to GC-MS analysis. Immediately after harvest, the tissues were weighed and rapidly frozen in liquid nitrogen. The frozen tissues were then ground into a powder and mixed with 500 μl of hexane in a glass tube. Methyl laurate (8.7 ng/μl, Sigma-Aldrich, Co., LLC., USA) was added as an internal standard. The mixture was vortexed for 30 seconds and sonicated for 5 minutes. The extracts were then centrifuged at 3000 g for 10 minutes, and the supernatant was dried using a column filled with Na_2_SO_4_. Subsequently, 1 μl of the hexane extract was injected in splitless mode into an Agilent 7890A GC coupled to an Agilent 5973 mass selective detector. A full scan from 33 to 500 amu was performed. The GC was equipped with an HP-5MS (Agilent Technologies, USA) capillary column (30 m × 0.25 mm i.d. × 0.25 μm). The GC oven temperature program started at 45°C, held for 2.25 minutes, then increased at a rate of 40°C per minute to 300°C with a 5-minute hold. The operating conditions were as follows: helium carrier gas with a constant flow rate of 1 ml/min and an injector temperature of 260°C. Mass spectra were obtained by electron impact at 70 eV. To identify the volatile compounds, the obtained mass spectra were compared to those in commercial and in-house mass spectral libraries (NIST14), and the retention indices were compared to those published in the literature. Retention indices were calculated based on a series of alkanes using a third-order polynomial function.

### 2.6 Headspace volatile collection of intact transgenic chrysanthemum plants

To analyze the quantity and composition of intact plant emitted volatiles, a dynamic headspace trapping system was employed. In order to minimize emissions from the soil, the plant pots were carefully wrapped in aluminum foil. The collection vessels used were sealed 10-liter glass containers equipped with two connector plugs on the top. Prior to introduction into the vessels, the air was passed through Teflon tubing and a charcoal filter to ensure its cleanliness, and it was then introduced at a controlled rate of 400 ml/min. The outlet of the system was connected to a glass GC inlet liner containing 1g of Tenax TA resin (60-80 mesh; SuperQ). For the mechanical damage treatment, flowering plants of the transgenic chrysanthemum were rapidly punctured 50 times with entomological pins on the flower stems and then immediately placed in the volatile collection vessels. Headspace collection was carried out for 24 hours, with 12 hours during the light period and 12 hours in darkness. To elute the volatiles from the liner, 1 ml of hexane (HPLC grade) containing methyl laurate (7.8 ng/μl, Sigma-Aldrich, Co., LLC., USA) as an internal standard was used. Finally, the samples were subjected to analysis by GC-MS using the same program employed for the analysis of hexane extraction of fresh tissues.

### 2.7 Histochemical staining

Fresh hand-sections were prepared from both the wild-type and transgenic Chrysanthemum ‘1581’ lines at the bud stage, and they were immediately treated with the NADI (naphthol + dimethyl paraphenylenediamine) reagent. The staining process followed the procedure described by Caissard et al (Caissard et al., 2004). Subsequently, the sections were directly observed using a microscope (Olympus BX53, Tokyo, Japan). For observing the sieve elements, fresh hand-sections were exposed to a solution of 0.1% aniline blue (Water Blue, Shanghai, China) in potassium phosphate buffer (0.1 M, pH 7.4) for 30 minutes. Afterward, they were rinsed in the same buffer for 10 minutes. Aniline blue fluorescence was examined using a fluorescence microscope (Olympus BX53) with an excitation light of 365 nm.

### 2.8 Aphid probing behavior

The feeding behavior of adult apterous cotton aphids was observed using a Giga-4-DC-EPG system (Tjallingii, Wageningen, the Netherlands). The aphids were monitored for their feeding activities on the flower stem of chrysanthemum plants, which were placed inside a Faraday cage in the laboratory with controlled conditions (20°C temperature, relative humidity between 60% and 70%). EPG recordings were conducted continuously for a duration of 8 hours, with a minimum of eight aphids observed per genotype. The Stylet+a software was utilized for recording and analyzing the data, and the waveforms were interpreted following the methodology described by Tjallingii (Tjallingii and Hogen Esch, 1993). The statistical analysis of the data was performed using the Mann-Whitney U-test.

### 2.9 Aphid olfactory dual-choice assay on detached flowers

We conducted a study to assess the response of aphids to olfactory cues using a dual-choice assay with detached flowers from both wild-type and transgenic plants, following the methodology described by Yang et al (Yang et al., 2013). Prior to bioassay, chrysanthemum leaves designated for rearing aphids were excised from the plants and placed in a glass bottle covered with gauze. This setup was maintained until the aphids left the feeding leaves. After one night of inoculation, cotton aphids (in the third or fourth nymphal stage) were meticulously transferred to a petri dish and subjected to a 4-hour period of starvation. We recorded the aphids from the stock rearing that selected a leaf within five minutes. The assay was replicated using a minimum of eight pairs of flowers, with each pair tested on at least ten individual aphids. For the mechanical damage treatment, flowers were punctured rapidly ten times using entomological pins on the flower receptacles. Subsequently, they were inserted into agar for five minutes before the start of the assay. In the assay, a metal wire (0.5 mm diameter, approximately 2.5 cm long) was positioned between two flowers (1.6 cm diameter), with their peduncles (1 cm) embedded in a 1.5% (w/v) agar bed in a 10 cm diameter Petri dish. One flower represented the control from wild-type chrysanthemum plants, while the other flower came from transgenic plants of either line PEE-2 or PEE-8, serving as the test. The metal wire did not come into direct contact with any of the flowers, maintaining a distance of approximately 1.5 cm between the wire end and the flowers. One aphid was released in the middle of the wire. After one or a few rounds of walking, the aphids eventually exited the wire at either end and proceeded towards their preferred flower. We recorded the number of aphids reaching each flower, and a χ2 test was employed to analyze the significant preference of aphids based on olfactory cues.

### 2.10 Statistical analysis

The assessment of EBF production and *EbFS* gene expression levels in transgenic chrysanthemum plants, in relation to wild-type plants, was presented as mean ± standard deviation (SD). Statistical analyses were performed utilizing ANOVA followed by multiple range test or two-tailed t-test, with a significance threshold set at P < 0.05. Results were based on at least three replicates from three independent experiments.

## 3 Results

### 3.1 Expression pattern analysis of the *TcEbFS* gene promoter in transgenic *C. morifolium*

The 2.2-kb promoter sequence of the *TcEbFS* gene (accession number deposited in GenBank: MF678596) was previously obtained from pyrethrum flower buds (Li et al., 2019). The transcript start site (TSS) was determined to be located 25 bp upstream of the predicted start codon and this was further confirmed by our 5’ RACE results of the *EbFS* cDNA sequence. The identified putative cis-acting regulatory elements are summarized in **Supplementary Table S1**. By comparing the *EbFS* promoter sequences in pyrethrum with its related species *Artemisia annua*, we observed both common and unique putative cis-acting regulatory elements in each promoter (**Supplementary Table S1**). Notably, the *EbFS* promoter region exhibited several motifs associated with plant hormone responses, including ethylene, abscisic acid (ABA), gibberellic acid (GA), jasmonic acid methyl ester (MeJA), salicylic acid (SA), and auxin. Additionally, motifs responsive to low temperature and light were also present within the promoter sequence (**Supplementary Table S1**).

**Figure 1:**
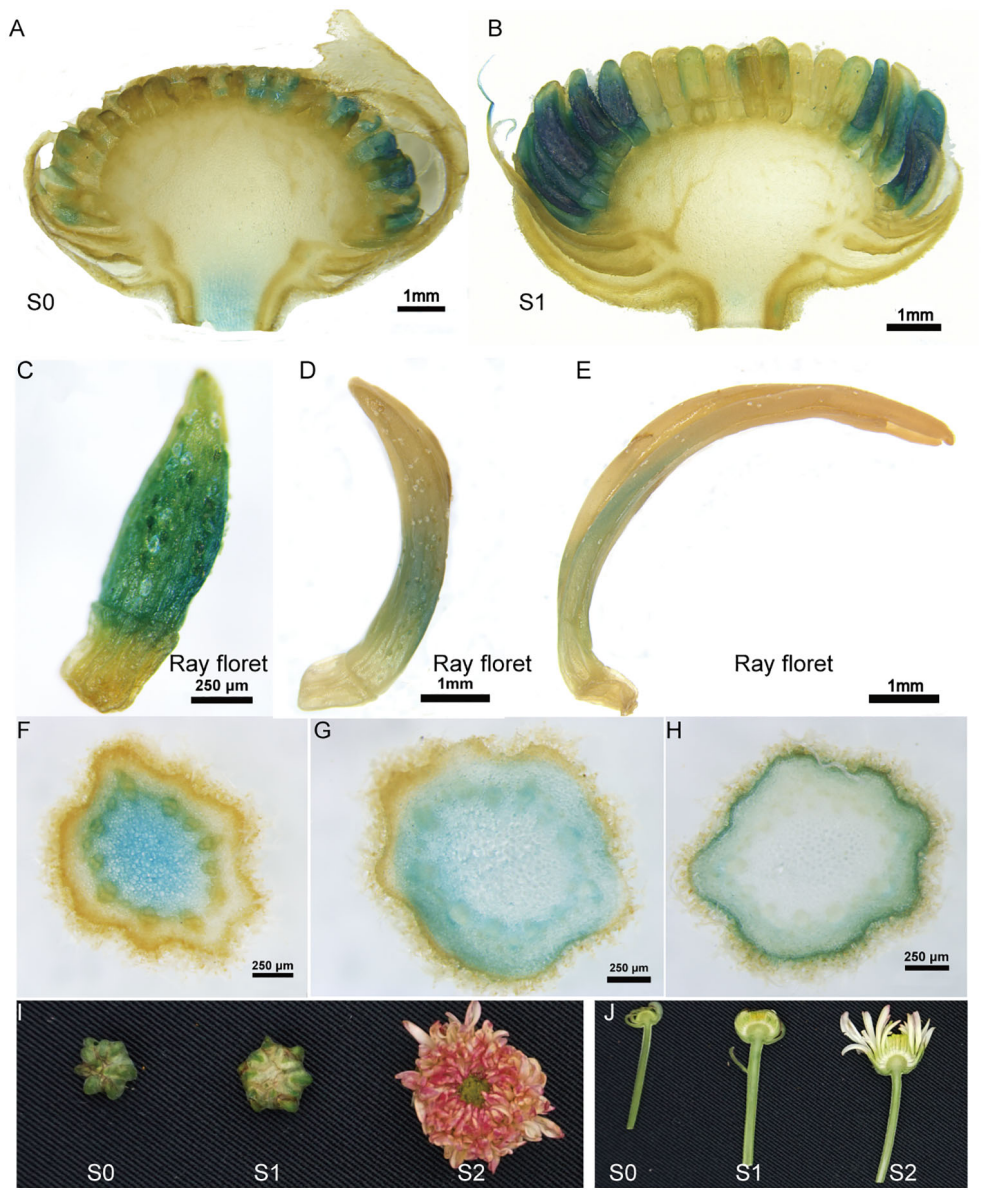
GUS staining of flower head and stem of *C. morifolium* carrying *pBI121-pE::GUS* vector. **(A, B)** GUS staining of longitudinal sections of S0 and S1 flower head; **(C)** GUS staining of the ray floret from the S0 flower; **(D)** GUS staining of the ray floret from the S1 flower; **(E)** GUS staining of the ray floret from the S2 flower; **(F–H)** GUS staining of cross sections of the upper, middle and lower flower stems of S1 flower; **(I)** pictures of chrysanthemum flower heads at different developmental stages; **(J)** longitudinal sections of chrysanthemum flowers attached to its stem.

To investigate the promoter activity in flower organs, GUS staining was performed on transgenic chrysanthemum flowers. High GUS expression was observed in the young flowers, but it notably decreased as the flowers developed to stage 2 with the erect ray florets, displaying a specific expression pattern in the ray florets (**Figures 1A–E**). Additionally, we observed variable expression patterns in different parts of the flower stems. The highest GUS expression was detected in the parenchyma cells within the pith of the upper stem, while significant GUS staining was observed in the cortex cells surrounding the vascular system in the lower stems (**Figures 1F–H**). The transgenic tobacco also exhibited specific expression of the *TcEbFS* gene promoter in the inner cortex cells and phloem along the vascular system (**Supplementary Figure S1**).

### 3.2 EBF emission and accumulation analysis in transgenic chrysanthemum carrying *pBI121-pE::EbFS* construct

**Figure 2:**
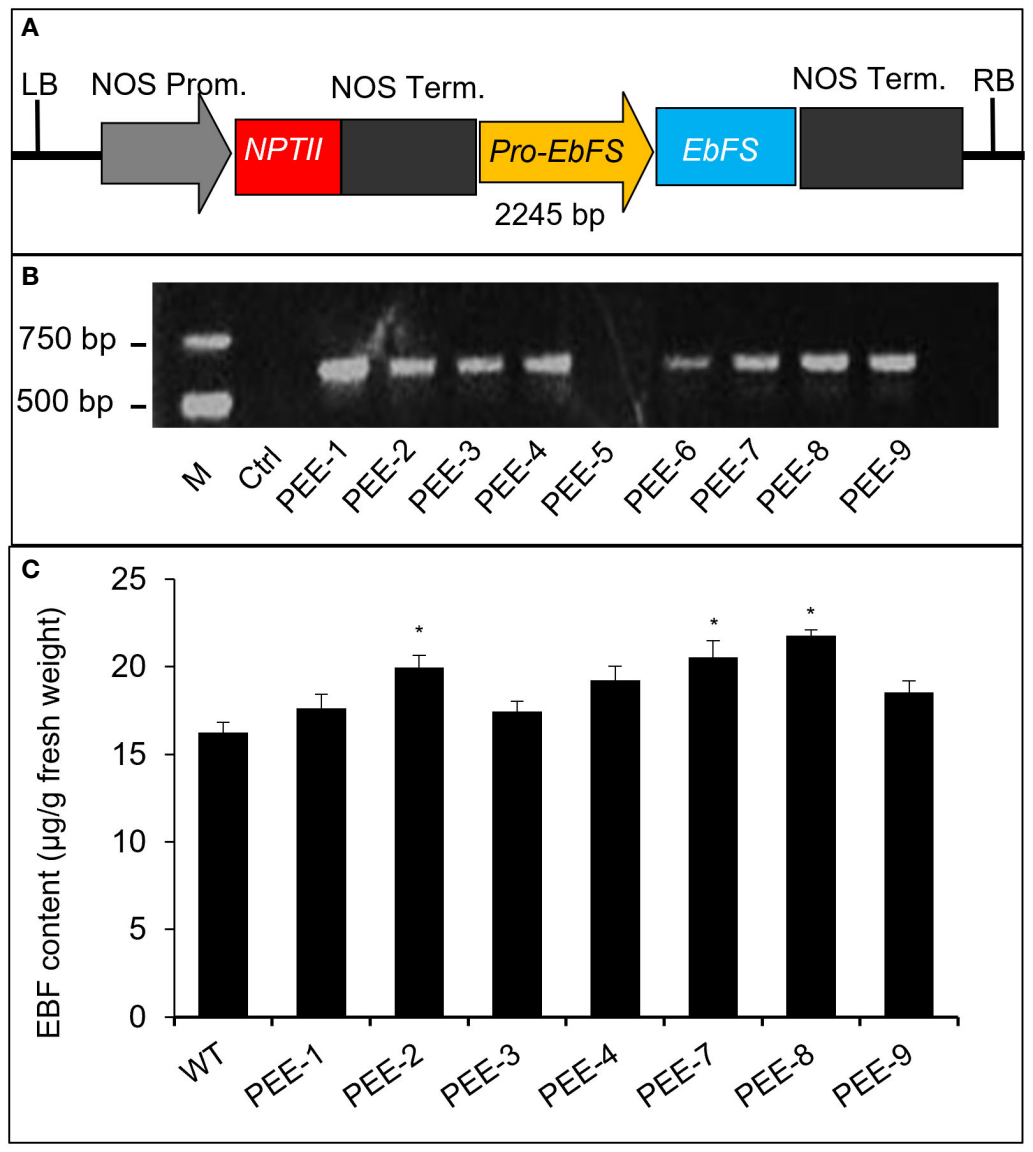
Transformation of the *TcEbFS* gene driven by its native promoter in *C. morifolium*. **(A)** schematic structure of *pBI121-pE:: EbFS* vector. **(B)** PCR identification of transgenic plants. Genomic DNA was amplified with the specific primers (P-2060F and *EbFS*-20R, listed in **Supplementary Table S2**). M, DNA marker; Ctrl, wild type chrysanthemum; PEE-1 - PEE-9, independent transgenic plants. **(C)** EBF content analysis of transgenic chrysanthemum plants. WT, wild type chrysanthemum. The asterisks indicate the significance of the EBF differences between transgenic plants and wild type (t-test: *, P ≤ 0.05).

**Figure 3:**
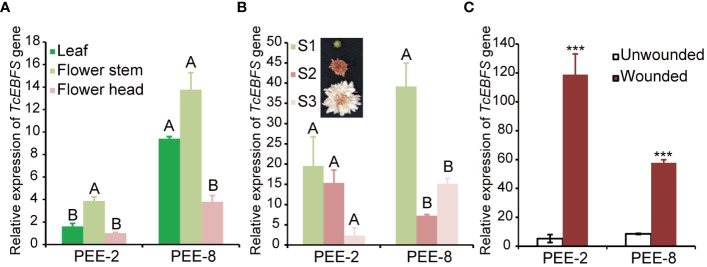
Relative expression of the *TcEbFS* gene in transgenic chrysanthemum. **(A)** expression analysis of the *TcEbFS* gene in different organs of transgenic chrysanthemum relative to the leaf (ANOVA followed by Duncan’s multiple range test. P < 0.01). **(B)** expression analysis of the *TcEbFS* gene in flower head at different developmental stages of transgenic chrysanthemum relative to S1 flowers (ANOVA followed by Duncan’s multiple range test. P < 0.01). **(C)** relative expression of the *TcEbFS* gene in flower stem of transgenic chrysanthemum upon mechanical damage. The wounded and unwounded flower stems (3 centimeters below the flower head) were individually sampled for gene expression analysis. The error bars represent ± SD of three biological replicates. The asterisks indicate the significance of the EBF differences between transgenic plants and wild type (t-test: ***, P ≤ 0.001).

Chrysanthemum plants are highly susceptible to aphid infestations, particularly during the flowering stages. To develop an aphid-resistant chrysanthemum variety, we introduced the pyrethrum *EbFS* gene, driven by its native promoter, into chrysanthemum (**Figure 2A**). From the resulting transgenic chrysanthemum plants, we selected two lines (line 2 and line 8) with the highest EBF accumulation in flower buds at stage 1 (S1) for further analysis (**Figure 2**). In comparison to the wild-type chrysanthemum, the overexpression of the *TcEbFS* gene did not manifest any discernible plant phenotypes (**Supplementary Figure S2**). Quantitative real-time PCR (qRT-PCR) analysis of *TcEbFS* gene expression in different organs of transgenic plants showed the highest *TcEbFS* expression in the flower stems of transgenic chrysanthemum (**Figure 3A**). Additionally, *TcEbFS* expression peaked in the S0 flower bud and exhibited a strong response to mechanical damage in transgenic chrysanthemum plants (**Figures 3B, C**).

**Figure 4:**
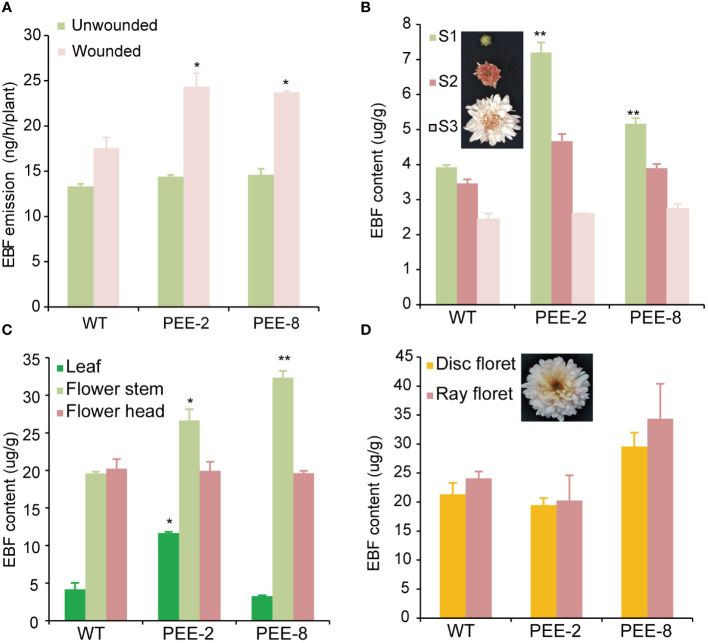
EBF content accumulated in the tissues of transgenic chrysanthemum plants compared to that of the wild type. **(A)** EBF emission from transgenic chrysanthemum flowering plants compared to wild type upon mechanical damage. Whole flowering plants of the transgenic chrysanthemum were wounded with entomological pins on the flower stems and then immediately placed in the volatile collection vessels. Volatiles were collected for a 24h day/night cycle and followed by GC-MS analysis. **(B)** analysis of EBF content in the leaves, flower heads and flower stems of S2 flowers. **(C)** analysis of EBF content in the flower head at different developmental stages (S1, flower bud; S2, flower with erect ray florets; S3, open flower with expanding ray florets and closed disc florets, as shown in the pictures). **(D)** EBF content individually accumulated in the disk and ray florets of S3 flowers. Disk and ray florets were dissected from S3 flowers and extracted with hexane. Error bars represent ± SD of three biological replicates. The asterisks indicate the significance of the EBF differences between transgenic plants and wild type (t-test: *, P ≤ 0.05; **, P ≤ 0.01).

To determine the volatile compound produced as a result of *TcEbFS* overexpression under its native promoter, we collected volatiles from intact flowering chrysanthemum plants using a dynamic headspace sampling system, followed by GC-MS analysis. During the 24-hour day/night collection period, no EBF emission was detected from the early flowering plants with dominant S0-S2 flowers. In pyrethrum, EBF is predominantly stored inside the flower and released mainly after the flower opening (particularly in the disc florets) or in response to mechanical damage (Li et al., 2019). Furthermore, we induced mechanical damage to the flower stems of flowering chrysanthemum plants with dominant S3 flowers and collected volatiles for GC-MS analysis. We observed significantly higher EBF emission (23-24 ng/h/plant) from the wounded transgenic chrysanthemum plants compared to the wild-type plants (17-18 ng/h/plant), however, no significant difference was discerned between these plants in the absence of damage (**Figure 4A**). This suggests that the increased EBF production in transgenic plants was not spontaneously released from the flower but rather induced by mechanical damage.

To determine the concentration of EBF accumulated within different plant tissues, we analyzed the solvent extraction of fresh leaf, flower head, and flower stem tissues at different developmental stages. For the flower head, only the S1 flower bud of transgenic chrysanthemum showed a significantly higher EBF content compared to the wild type (**Figure 4B**). However, EBF content was highest in the flower stem of both transgenic chrysanthemum lines, which differed from the original EBF accumulation pattern in the wild type (**Figure 4C**). Additionally, we analyzed the hexane extraction of the disc florets and ray florets individually, considering the abundant expression of the *TcEbFS* gene promoter in the ray florets. More EBF was found to accumulate in the ray florets of both the wild type and transgenic plants, with higher levels observed in the PEE-8 line (**Figure 4D**).

### 3.3 Localization of terpene accumulation in the chrysanthemum flower

**Figure 5:**
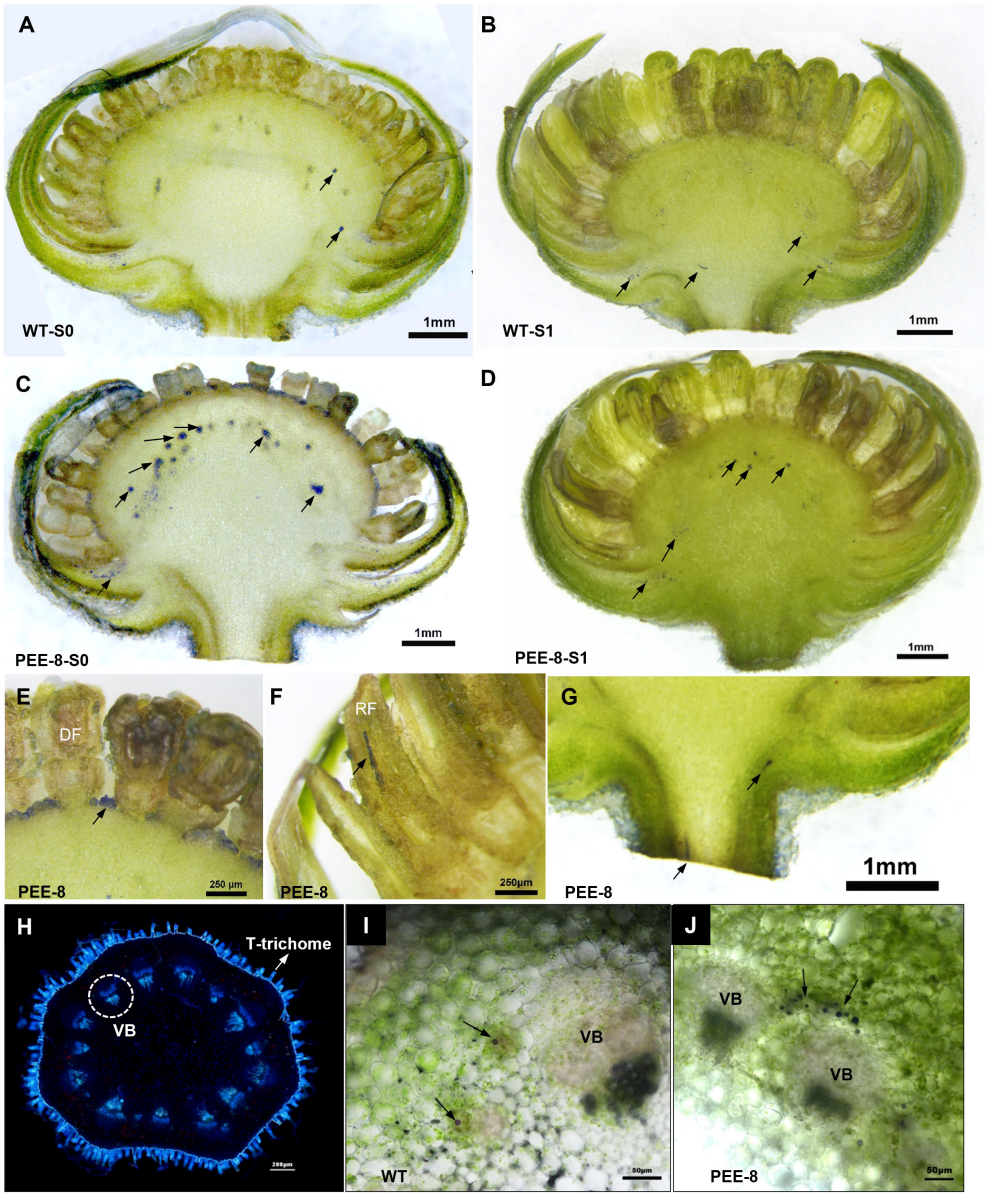
Terpenes localization in the flower heads and flower stems of wild-type and transgenic chrysanthemum. **(A–G)** NADI staining of longitudinal sections of S0 (younger flower bud) and S1 flower heads of wild-type and PEE-8 transgenic chrysanthemum, respectively. **(H)** aniline blue staining of cross sections of flower stems of wild-type chrysanthemum showing the vascular bundle with stained callose of sieve plates. **(I, J)** NADI staining of cross sections of S1 flower stems of wild-type and PEE-8 transgenic chrysanthemum, respectively. The black arrows point to the stained terpene oils. CGT, capitate glandular trichome; VB, vascular bundle.

To investigate the accumulation of terpenes, particularly EBF, in the cortex cells where the *TcEbFS* promoter was specifically expressed, we conducted NADI (naphthol + dimethyl paraphenylenediamine) staining, which specifically stains terpene oils (Li et al., 2019). The staining revealed that terpene oils were predominantly accumulated in the receptacle of both transgenic and wild-type chrysanthemum, but the transgenic plants exhibited a significantly higher abundance of stained oil droplets, particularly in the S0 flower head (**Figures 5A–D**). In transgenic chrysanthemum, stained terpene oils were also observed in the base of the single floret and the upper part of the ray floret (**Figures 5E, F**). Interestingly, we observed that the chrysanthemum flower stem was densely covered with T-shaped non-glandular trichomes, as revealed by aniline blue staining for sieve elements. This trichome distribution was distinct from the flower stem of pyrethrum (**Figure 5H**). In pyrethrum, abundant secretory ducts along with the vascular bundles are responsible for storing cortex-produced EBF (Li et al., 2019). In chrysanthemum flowers, sporadic purple-stained terpene oil droplets or oil stripes were observed in the cortex cells around the vascular bundles, with a significantly higher abundance in the transgenic chrysanthemum plants (**Figures 5G, I, J**).

### 3.4 Behavior analysis of aphids on transgenic and wild-type chrysanthemum

**Figure 6:**
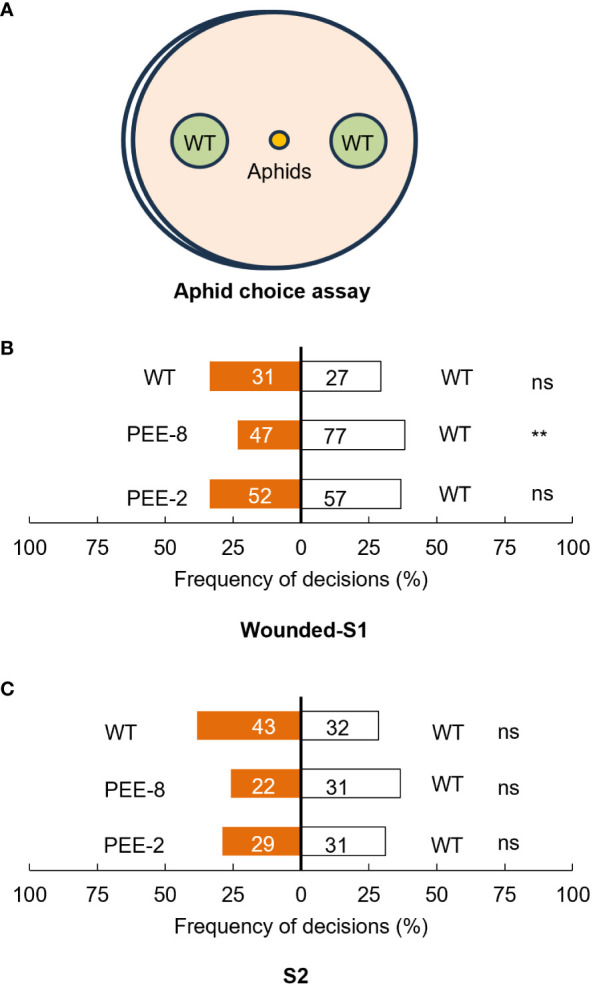
Choice behavior assay of aphids for wounded S1 and intact S2 flowers of transgenic and wild type chrysanthemum plants. **(A)** Illustration of aphid choice behavior experiment. **(B)** Olfactometer choices of cotton aphids in response to wounded S1 of transgenic and wild type chrysanthemum plants. **(C)** Olfactometer choices of cotton aphids in response to intact S2 flowers of transgenic and wild type chrysanthemum plants (paired t-test, **, P < 0.01; ns, not significant). Each bars represent the percentage of aphids that made a choice within 5 minutes after release. The number on the bar represent number of aphids in total.

Plants commonly release EBF as a defense signal to deter aphids, prompting us to investigate aphid preference for flowers of transgenic chrysanthemum compared to wild-type plants in a dual-choice assay solely based on olfactory cues (Hu et al., 2018). As the emission of EBF was only significantly induced after mechanical damage, we also included wounded S1 flowers in the assay. Within 5 minutes, nearly 60% of aphids made a selection, but significant repellent effect was only observed for wounded transgenic flowers (**Figure 6**). To gain further insight into the feeding behavior of cotton aphids, we employed the electrical penetration graph (EPG) technique to monitor their probing and feeding activities when feeding on transgenic and wild-type chrysanthemum plants. It was observed that on both transgenic chrysanthemum lines, the initial probing time was 2-3 times longer. Notably, the duration and frequency of salivation in the phloem phase (E1) were higher on transgenic plants (**Table 1**). Specifically, the mean probing time was significantly higher for the transgenic chrysanthemum PEE-8 line compared to the wild type (**Table 1**). These findings collectively indicate a disruption in the host plant location behavior of aphids on transgenic plants.

**Table 1:** EPG parameters of cotton aphids (*Aphis gossypii*) during an 8-h recording on flower stem of chrysanthemum plants.

EPG parameters	WT (n=8)	PEE-2 (n=11)	PEE-8 (n=8)
Total duration of non-probing (h)	2.42 ± 0.37	2.52 ± 0.35	1.14 ± 0.27**
Duration of first probe (min)	1.51 ± 0.29	4.58 ± 2.93	3.92 ± 2.14
Number of probes	14.88 ± 2.04	12.27 ± 1.37	10.13 ± 1.40*
Total duration of probing (h)	2.62 ± 0.18	3.21 ± 0.56	2.93 ± 0.34
Mean of probing	0.21 ± 0.04	0.27 ± 0.04	0.34 ± 0.06*
Number of probes to the first E1	10.00 ± 1.40	8.18 ± 1.71	7.13 ± 1.69
Duration of probing period before the first E1 (h)	1.85 ± 0.28	2.17 ± 0.75	1.62 ± 0.54
Number of E1	1.88 ± 0.38	2.09 ± 0.66	2.43 ± 0.51
Total duration of E1 (min)	19.66 ± 13.47	31.14 ± 20.58	24.77 ± 13.36
Mean of E1	0.28 ± 0.23	0.12 ± 0.07	0.14 ± 0.07
Number of E2	1.38 ± 0.40	1.27 ± 0.40	2.50 ± 0.93
Total duration of E2 (h)	1.45 ± 0.38	0.97 ± 0.36	2.21 ± 0.57
Mean of E2	1.18 ± 0.39	0.69 ± 0.31	0.95 ± 0.31
Number of G	0.63 ± 0.31	0.36 ± 0.31	0.50 ± 0.36
Duration of G (h)	0.39 ± 0.21	0.15 ± 0.13	0.26 ± 0.19
Phloem phase index^1^	0.50 ± 0.13	0.29 ± 0.09	0.40 ± 0.09
Phloem salivation index^2^	0.20 ± 0.12	0.20 ± 0.09	0.23 ± 0.12

E1, salivation phase; E2, phloem sap ingestion; G, xylem ingestion. n, number of replicates.

^1^Index calculated as: E1+E2/(C+E1+E2+G+F).

^2^Index calculated as: E1/(E1+E2).

## 4 Discussion

Our investigations revealed that in both pyrethrum and transgenic chrysanthemum, EBF is synthesized in the cortex cells and pith (**Figures 1**, **5**). However, in pyrethrum, EBF is subsequently transported and stored in secretory cavities/ducts around the vascular bundles, particularly during the disintegration of the pith (Li et al., 2021). This production and storage pattern has two important implications for their defensive function. Firstly, aphids extensively probe and release saliva into potential host plants, often rejecting non-hosts after initial probing of epidermal cell contents, or subsequent probing of mesophyll cell contents (Züst and Agrawal, 2016). Certain terpenes that accumulate in mesophyll cells can render plants detectable to foraging aphids (Dancewicz et al., 2016). The longer probing time observed on transgenic chrysanthemum likely indicates that EBF accumulation serves as a deterrent function (**Figure 6**). Secondly, the secretory cavities/ducts in the flowers serve as conduits for storing and transporting EBF to the flower stigma and corolla of the open disc florets for subsequent emission (Li et al., 2021). While the pyrethrum flower head is supported by a long peduncle rising from the plant’s base and consists of an outer ring of white ray florets and a yellow flower heart densely populated by disc florets in the center of the receptacle (Li et al., 2021). In chrysanthemum species, the emission rates of floral terpenes largely depend on their internal concentrations, and the disc florets have been found to contribute more to floral volatile emission than ray florets (Zhang et al., 2021). However, in our chrysanthemum cultivar ‘1581’, ray florets constitute nearly 80-90% of the entire flower head. We observed specific expression of the *TcEbFS* gene promoter and slightly higher EBF accumulation in the ray florets, which contrasts with the high expression and accumulation in disc florets of pyrethrum (**Figures 1**, **4D**) (Li et al., 2021). This finding likely constrains the EBF accumulation and emphasizes the significance of the spatial distribution, translocation, and storage of specific volatiles in different organs and tissues, which are crucial for their release from floral organs at specific stages of flower development, from specific cells, and at specific times of the day (Tissier et al., 2017; Turner et al., 2018; Bouwmeester, 2019). Therefore, in future studies, it is important to consider the dedicated organs or tissues where many of these metabolites are synthesized and accumulated, such as specialized structures located on the surface (glandular trichomes) or internally (secretory ducts/cavities) in the flower, as well as the ratio of ray and disc florets when genetically engineering specialized metabolites in other related chrysanthemum species.

The strong induction of the *TcEbFS* gene by mechanical damage in transgenic chrysanthemum is evident in **Figure 4C**. Numerous studies have illustrated that mechanical damage initiates the transcription of terpene synthase genes in various plant species (Wang et al., 2013; Li et al., 2014). Mechanical damage serves as a complex stress signal, often associated with herbivore attacks or pathogen infestations. In pyrethrum, any environmental stress causing mesophyll cell rupture triggers a swift transcriptional response of the *EbFS* gene, leading to the release of EBF (Li et al., 2019). The presence of the damage-inducible *TcEbFS* gene in transgenic chrysanthemum suggests its potential role in establishing an aphid defense strategy.

It is important to note that in pyrethrum, EBF accumulates in the pyrethrum flower peduncle in a nearly pure form, at concentrations almost ten times higher than that found in transgenic chrysanthemum (**Figure 4**) (Li et al., 2019, 2021). This high and pure accumulation leads to the excretion of EBF in aphid honeydew because the cortex cells, which are rich in EBF oils and surround the phloem tissue, are occasionally sampled by aphids during the probing phase in search of the phloem sieve elements. These high concentrations of EBF induce aphid alarm responses and may represent an additional, more intimate level of flower defense. In this study, increased EBF emission from wounded flowers shows significant repellence to aphids, moreover the probing time of aphids on transgenic chrysanthemum is much higher than wild type plants (**Table 1**). As demonstrated by Vargas et al. (2005), polyphagous aphids heavily rely on gustatory stimuli for host selection (Vargas et al., 2005). The study concluded that olfactory signals did not significantly influence host selection by *Myzus persicae*. However, this aphid species recognized its host plant following a brief probe of subepidermal tissues.

Numerous studies have endeavored to enhance plant insect resistance through the modification of specific terpene profiles. However, the efficacy of terpenoid engineering in plants is constrained by several factors: a) availability of both the substrate pool and the incorporated branch point enzyme in the same sub-cellular compartment. To prevent autotoxicity, plants typically suppress the biosynthesis of these terpenes in these specific tissues due to the restricted availability of substrates. It is speculated that the physical separation of EBF production in the cortex cells from other terpenes derived from glandular trichomes may play a crucial role in remaining the high purity and dominance of EBF in pyrethrum flowers. In chrysanthemum, despite our NADI staining assay indicating a higher terpene presence in the cortex cells, no internal secretory structures responsible for subsequent terpene storage were discerned (**Figure 5**). b) the specific activity of endogenous enzymes if any sharing the same substrate. In our analysis of chrysanthemum flower head and stem extracts, we detected abundant monoterpenes and sesquiterpenes, which are often synthesized and stored in glandular trichomes (Guo et al., 2020; Guan et al., 2022) (**Supplementary Figure S3**, **Supplementary Data S1**). In pyrethrum, a subset of terpene-based end-products is synthesized in the epidermal glandular trichomes and then transported to subepidermal tissues for further synthesis or storage. The interconnectedness of these two secretory structures allows for the directional translocation of these metabolites to various destinations. In chrysanthemum, EBF constitutes a minor component within the complex blend of terpenes. Apart from EBF, our analysis also revealed higher accumulation of several other terpenes, including camphene, phellandrene, and 2-carene, among others, in the flower stems of transgenic chrysanthemum (Data S1). This observation suggests that other terpene synthases with high activities may be concurrently utilizing the same pool of substrates (Jiang et al., 2021). As demonstrated in pyrethrum, the high and pure accumulation of EBF is a critical factor for establishing aphid defense function. It remains quite challenging to attribute changes in aphid behavior solely to a specific compound, especially in chrysanthemum, which is renowned for its rich accumulation of terpenes. While exogenously applied camphene may disrupt aphid recognition and acceptance, our understanding of the biological function of individual compounds remains incomplete when they are blended within such an abundant terpene background (Dancewicz et al., 2016). However, we cannot exclude the possibility that these compounds may contribute to the observed changes in aphid behavior in transgenic plants. c) the costs of engineering in terms of effects on general growth and physiology of the plant as the substrate is common for many downstream end-products. Same as illustrated in Hu et al. (2018), the transgenic introduction of a terpene synthase gene into chrysanthemum ‘1581’ did not manifest in discernible phenotypic alterations in growth and development (Hu et al., 2018) (**Supplementary Figure S2**). In contrast, Yang et al. reported that the incorporation of a linalool synthase gene under the control of the Rubisco small subunit promoter led to slightly reduced leaf length and a lighter leaf color, implying that the influence on phenotypes could be contingent on the specific metabolites engineered (Yang et al., 2013). If pivotal metabolic pathways are impacted, it is probable to induce substantial changes in phenotype.

This study represents a significant advancement in demonstrating the stable genetic engineering of pheromone biosynthesis in chrysanthemum. However, further investigations are required to fully understand the underlying mechanisms. Firstly, it is crucial to determine whether the secretory structures in chrysanthemum are responsible for the translocation and storage of terpenes. Secondly, the conflict in substrate flux towards EBF and other abundant terpenes needs to be addressed. Additionally, the overall outcome of volatile semiochemical production under specific pressures, such as herbivore attack, should be comprehensively considered. Future efforts should focus on harnessing this inherent metabolic engineering capacity to develop the production of defensive compounds in plants.

## 5 Conclusion

In this study, our aim was to introduce the defensive mimicry system of the pyrethrum plant into chrysanthemum flowers for aphid resistance. We successfully achieved high and specific expression of the *TcEbFS* gene promoter in transgenic tobacco plants and chrysanthemum flowers, resulting in relatively higher accumulation of EBF in the chrysanthemum flower stem and young flower bud. Interestingly, we observed prolonged probing phases by aphids on the flower stems of transgenic chrysanthemums. Coupled with the finding that a significant repellent effect against aphids was only evident in the wounded S1 transgenic flowers, our results suggest that the targeted accumulation of EBF oils might influence aphid host location, albeit only repelling them when higher levels of EBF are released upon damage. This finding suggests that the transgenic application of the defensive mimicry strategy in other plants may be challenging. It highlights the complexity of plant defense mechanisms and the need to understand how pyrethrum has evolved to achieve its highly effective defensive benefit.

## Data availability statement

The datasets presented in this study can be found in online repositories. The names of the repository/repositories and accession number(s) can be found in the article/**Supplementary Material**.

## Author contributions

JJL: Writing – original draft, Writing – review & editing. HH: Writing – review & editing. SR: Investigation, Writing – review & editing. LY: Data curation, Writing – review & editing. YL: Methodology, Writing – review & editing. JWL: Investigation, Writing – review & editing. TZ: Methodology, Writing – review & editing. MW: Writing – review & editing. CW: Writing – review & editing, Supervision.
