# Supplementary material for: Aphid alarm pheromone mimicry in transgenic Chrysanthemum morifolium: insights into the potential of (E)-β-farnesene for aphid resistance
Source: Front Plant Sci. 2024 Apr 22;15:1373669. doi: 10.3389/fpls.2024.1373669 (PMC11070518; doi:10.3389/fpls.2024.1373669)
Supplement: Supplementary file 1 [file DataSheet_1.docx]

Supplementary Material


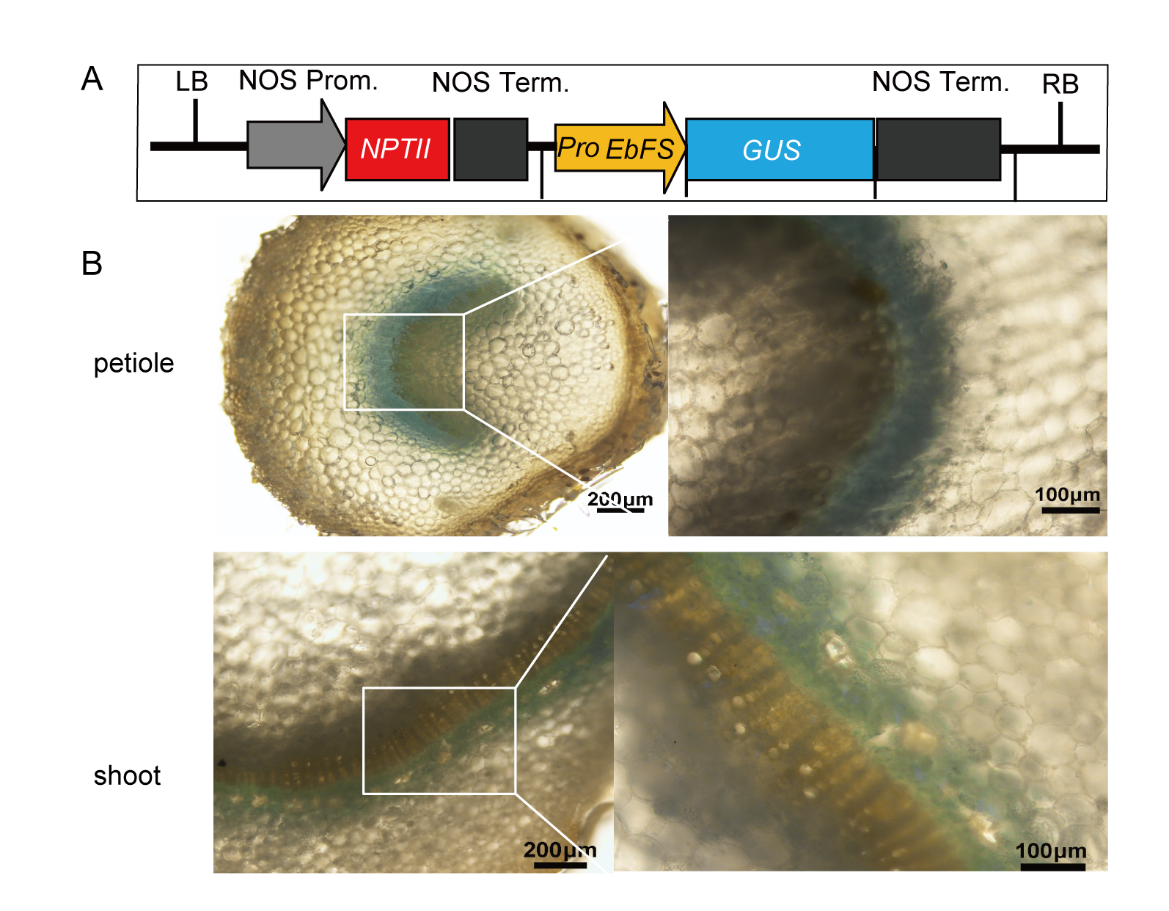


**Supplementary Figure 1. Tissue-specific GUS activity analysis in transgenic plants of *N. tabacum* carrying *pBI121-pE::GUS* vector.** A, schematic structure of *pBI121-pE::GUS* vector. B, histochemical localization of GUS activity was specifically observed in the inner cortex cells and phloem adjacent to the vascular system.

**Supplementary Figure 2. Representative photographs of flowers and leaves isolated from transgenic and wild-type chrysanthemum plants.**

**Supplementary Figure 3. Analysis by GC-MS of volatile compounds of chrysanthemum plants.** A, typical GC-MS chromatograms showing the hexane extracts of S2 flower heads of intact wild type chrysanthemum plant and transgenic chrysanthemum plant at RT 5.5 - 20 min. B, typical GC-MS chromatograms showing the hexane extracts of flower stems of intact wild type chrysanthemum plant and transgenic chrysanthemum plant at RT 5.5 - 20 min. IS, internal standard (methyl laurate). RT, retention time.

**Supplementary table 1. Number of putative cis-acting regulatory elements in promoter region of (*E*)-beta-farnesene synthase genes from *A. annua* and *T. cinerariifolium*.**

| Cis-acting  elements | Sequences | Promoter | | Description |
| --- | --- | --- | --- | --- |
|  |  | Artemisia | Pyrethrum |  |
| ABRE | ACGTG | 0 | 5 | Cis-acting element involved in the abscisic acid responsiveness |
| ACE | AAACCGGTTA | 1 | 0 | Cis-acting element involved in light responsiveness |
| ARE | AAACCA | 3 | 1 | Cis-acting regulatory element essential for the anaerobic induction |
| Box I | TTTCAAA | 4 | 1 | Light responsive element |
| Box 4 | ATTAAT | 3 | 3 | Part of a conserved DNA module involved in light responsiveness |
| CAT-box | GCCACT | 1 | 1 | Cis-acting regulatory element related to meristem expression |
| CGTCA-motif | CGTCA | 0 | 5 | Cis-acting regulatory element involved in the MeJA-responsiveness |
| ERE | ATTTTAAA | 0 | 1 | Ethylene-responsive element |
| G-box | CAC(G)GTC | 3 | 5 | Cis-acting regulatory element involved in light responsiveness |
| GCN4-motif | TGAGTCA | 1 | 1 | Cis-regulatory element involved in endosperm expression |
| LTR | CCGAAA | 0 | 3 | Cis-acting element involved in low-temperature responsiveness |
| O_2_-site | GATGACATGG/GTTGACGTGA | 0 | 2 | Cis-acting regulatory element involved in zein metabolism regulation |
| TATC-box | TATCCCA | 0 | 1 | Cis-acting element involved in gibberellin-responsiveness |
| TC-rich repeats | GTTTTCTTAC/ATTCTCTAAC | 3 | 2 | Cis-acting element involved in defense and stress responsiveness |
| TCA-element | CCATCTTTTT | 0 | 1 | Cis-acting element involved in salicylic acid responsiveness |
| TGA-element | AACGAC | 0 | 1 | Auxin-responsive element |
| chs-CMA2a | TCACTTGA | 0 | 1 | Part of a light responsive element |
| chs-CMA1a | TTACTTAA | 1 | 0 | Part of a light responsive element |
| Skn-1-motif | GTCAT | 3 | 7 | Cis-acting regulatory element required for endosperm expression |
| Circadian | CAANNNNATC | 0 | 1 | Cis-acting regulatory element involved in circadian control |

Supplementary table 2. FP primers (no hair pin structure), universal primers and specific primers used for cloning of the EbFS gene promoter. Primers used for the vector constructs were shown in the table.

| Name | Primer sequence 5’-3’ | Primer use |
| --- | --- | --- |
| pro*EbFS*-121-F | GACCATGATTACGCCAAGCTTATGGGTTGGTCCACTGGTTGAG | *pBI121-pE::GUS* |
| pro*EbFS*-121-R | GGACTGACCACCCGGGGATCCCTTCTCAAAATCTTGCAAGTTTGAT | *pBI121-pE::GUS* |
| pBI121-F | GATTATGAAGGACGTGCAAC | Constructs detection |
| pBI121-GUS-R | GGCGAACTGATCGTTAAAACTGC | Constructs detection |
| P-2060F | GATTATGAAGGACGTGCAAC | Constructs detection |
| *EbFS*-20R | CGTAACCAAATCTTGTGGCTCA | Constructs detection |
| pro*EbFS*-121-*EbFS*-F | CAAGATTTTGAGAAGGATCCATGTCAGCTATTCCTGTTTCTGGTG | *pBI121-pE::EbFS* |
| pro*EbFS*-121-*EbFS*-R | CGATCGGGGAAATTCGAGCTCTTAGACAACCATAGGGTGAACGAAG | *pBI121-pE:: EbFS* |
| *CmUBI*-F | AGCTGAGCAGACTCCCGATG | Real time PCR |
| *CmUBI*-R | AGGCGAATCATCAGTACCAAGT | Real time PCR |
| *EbFS* -F | CTACAGCTGTCCCATTTCCACTCC | Real time PCR |
| *EbFS* -R | CATGTAACTTATCATTTCTTCTC | Real time PCR |
